# Functional recovery and domain-specific quality of life after moderate-to-severe traumatic brain injury: a mixed-effects analysis

**DOI:** 10.3389/fneur.2026.1871168

**Published:** 2026-10-27

**Authors:** Ramzi Badra, Brooke Scardina, Mason Nelson, Varun Mishra, Taylor P. van Doren, Melissa Martinez, Justin L. Weppner

**Affiliations:** 1 Edward Via College of Osteopathic Medicine, Blacksburg, VA, United States; 2 Virginia Tech-Carilion School of Medicine, Roanoke, VA, United States; 3 High Point University, High Point, NC, United States; 4 Carilion Clinic Brain Injury Center, Roanoke, VA, United States

**Keywords:** functional recovery, Glasgow outcome scale extended, patient-reported outcomes, quality of life, traumatic brain injury

## Abstract

**Introduction:**

The purpose of this study is to investigate how functional recovery, measured by the Glasgow Outcome Scale-Extended (GOSE), is associated with quality of life (QOL) as measured by the Quality of Life after Brain Injury (QOLIBRI) survey in adults with moderate-to-severe TBI (msTBI). We aim to better understand this nuanced relationship to guide more individualized and effective rehabilitation planning.

**Methods:**

This study enrolled 138 adults aged 18 to 65 years with a history of msTBI one-year post-injury. Functional recovery was assessed using the GOSE score, administered face-to-face by a single trained investigator. Health-related QOL was evaluated using the QOLIBRI across six domains: Emotion, Feeling, Independence, Physical, Social, and Thinking. A mixed effects model using participant as the primary random effect and estimated marginal trends were calculated to examine the relationship between GOSE scores and QOLIBRI outcomes.

**Results:**

GOSE score alone is not significantly associated with QOL. Estimated marginal trends showed that higher GOSE scores were strongly associated with better QOL in the Independence (*p* < 0.001), Thinking (*p* < 0.001), and Physical (*p* = 0.019) domains. A weak, non-significant association was observed with the Feelings domain. No significant association was found between GOSE scores and the Social domain.

**Conclusion:**

Functional recovery after TBI is most closely related to independence, cognitive, and physical QOL but does not adequately reflect social or emotional well-being. These psychosocial domains may require targeted intervention and further emphasizes the importance of incorporating patient-reported QOL measures to more holistically assess recovery.

## Introduction

Traumatic brain injury (TBI) affects a large and growing global population, with the Global Burden of Disease analysis estimating approximately 20.84 million incident cases, 37.93 million prevalent cases, and 5.48 million years lived with disability worldwide (1). TBI survivors often report persistent physical symptoms, including headaches, nausea, fatigue, dizziness, lethargy, and sleep disturbances. These physical symptoms are often superimposed on cognitive and affective disturbances. Many TBI survivors report trouble with memory, concentration, and attention while others experience personality changes, depression, and anxiety following injury (2).

The Glasgow Outcome Scale–Extended (GOSE) is the most widely used global measure of functional recovery following TBI (3). Due to widespread adoption of this scale the GOSE score has been recognized as a core outcome metric by the National Institute of Neurological Disorders and Stroke (3). GOSE is administered as a structured interview where the examiner asks a series of questions pertaining to abilities related to basic and instrumental activities of daily living and social functioning (4). The scale is comprised of eight levels of performance after TBI: (1) dead; (2) vegetative state; (3) lower severe disability; (4) upper severe disability; (5) lower moderate disability; (6) upper moderate disability; (7) lower good recovery; and (8) upper good recovery (3). Strengths of GOSE are that it emphasizes daily functioning, is straightforward to interpret, and the ability to administer via multiple modalities such as in-person, phone, or mail (5). However, patients can be misclassified with GOSE and there is a lack of a true gold-standard method for administration (5). In addition, borderline cases exist and can be challenging to assign a rating (4). While useful for primarily capturing functional independence and disability, GOSE does not fully reflect patients’ lived experiences or perceived health-related quality of life (QOL).

To address this limitation, clinicians and researchers increasingly incorporate QOL measures such as the Quality of Life after Brain Injury (QOLIBRI) questionnaire, a patient-reported outcome measure designed to evaluate health-related QOL across six domains central to recovery (as they appear on the questionnaire): “Thinking abilities,” “Emotions,” “Independence,” “Social relationships,” “Feelings,” and “Physical problems” (6). The “Emotions” domain captures psychiatric disturbances such as depression, anxiety, and agitation, which are common after TBI and significantly reduce QOL. The “Feelings” domain assesses changes in self-perception and identity, often seen after neurological injury. The “Independence” domain measures the ability to perform daily activities and make decisions, reflecting key rehabilitation outcomes. The “Physical problems” domain evaluates symptoms like dizziness, headaches, and fatigue, which can complicate and prolong recovery. The “Social relationships” domain addresses interpersonal relationships and community participation, both essential for long-term recovery and reintegration. Finally, the “Thinking abilities” domain reflects impairments in memory, concentration, and attention, which are directly associated with academic and occupational outcomes. Together, these domains provide a comprehensive framework to understand the lived experiences of TBI survivors and guide clinical intervention (6).

As TBI research continues to evolve, there has been a growing recognition of the need to assess the patients’ own perspectives on QOL to fully understand recovery following TBI (6–8). Functional scales, such as the GOSE, remain the cornerstone of TBI outcome measurement, providing standardized classification of disability and independence. Yet, large-scale prospective studies such as TRACK-TBI have shown that even years after injury, many survivors continue to experience substantial symptom burden and reduced QOL, often irrespective of their functional recovery status (9). This disconnect highlights an important gap: functional recovery does not always equate to meaningful recovery from the patient’s perspective.

Prior work has demonstrated moderate-to-strong associations between GOSE and health-related QOL (3, 10). However, these studies have largely treated QOL as a single construct, without evaluation of how each specific domain is related to functional recovery or accounting for demographic and injury-related factors. The present study addresses these gaps by investigating whether GOSE scores is associated with QOL as measured by the QOLIBRI in adults with moderate-to-severe TBI (msTBI). By adopting a domain-specific analytic approach and adjusting for key variables, this study seeks to clarify how functional recovery translates into patient-perceived QOL. Understanding these complex relationships may ultimately guide more individualized rehabilitation planning and enhance patient-centered outcome assessment.

## Methods

### Participants

We enrolled 138 adults aged 18 to 65 years with a history of msTBI at least one-year post-injury. Eligibility was defined by a best Glasgow Coma Scale (GCS) score of 3–12 within the first 24 h, loss of consciousness lasting more than 30 min, or post-traumatic amnesia exceeding 24 h. Participants were classified as having moderate TBI (GCS score of 9–12, LOC of 30 min to 24 h, and/or PTA of 1–7 days) or severe TBI (GCS score of 3–8, LOC exceeding 24 h, and/or PTA exceeding 7 days). Participants were excluded if they had a significant preexisting neurological condition prior to their TBI that could confound the assessment. All eligible individuals approached for participation consented and completed the self-reported measures; there were no refusals, withdrawals, or incomplete assessments among approached participants. All participants completed both the GOSE score and QOLIBRI assessments. Individuals with GOSE scores of 1 or 2 (death or vegetative state) were excluded due to inability to complete self-reported measures. Written informed consent was obtained, and the study was approved by the Institutional Review Board at the institution that the research was performed (IRB Protocol 24–1932). The average age was 35 years (SD 14.0), including 110 males, 112 severe cases, with the three most common mechanisms of injury being motor vehicle collisions (*n* = 72), falls (*n* = 26), and blunt traumatic injury (*n* = 26). The mean GOSE score was 6.3 (SD 1.6), with a median of 7 and a range of 3 to 8. Table 1 reports descriptive statistics of the participants and the variables assessed.

**Table 1:** Descriptive statistics of continuous and categorical variables used to analyze the relationship between QOL and GOSE score.

Variable	Descriptive statistics		
Continuous variables	Mean (SD)	Median	Range
Age	35.0 (14.0)	30	18–65
GOSE score	6.3 (1.6)	7	3–8
Total QOLIBRI score^1^	63.8 (9.3)	64	41–89
QOLIBRI by domain^1^			
Emotions	42.7 (23.2)	43	0–100
Feelings	47.8 (24.9)	45	0–100
Independence	82.9 (21.3)	90	22.5–100
Physical problems	76.7 (14.3)	75	25–100
Social relationships	65.1 (19.4)	68	7.5–100
Thinking abilities	67.2 (27.0)	68	0–100

Categorical variables	Frequency	%
Sex		
Female	28	20.3
Male	110	79.7
Severity of TBI		
Moderate	26	18.8
Severe	112	81.2

Mean, standard deviation, median, and range of continuous variables are reported, while frequency and percent composition of each categorical variable are reported.

^1^Total QOLIBRI score and QOLIBRI score by domain are the standardized scores out of 100.

### Measures

#### GOSE

In this study, a single investigator was responsible for administering the GOSE, thereby eliminating interrater variability. The GOSE interview was conducted face-to-face. Prior to data collection, the investigator completed a training program and received a comprehensive study manual, which included guidance on handling borderline cases, challenging scenarios, and scoring procedures.

The GOSE questionnaire comprises 14 items organized into 7 sections, mirroring the structure of the interview. It is designed for use with adult patients or their caregivers. The questionnaire text is written at a Flesch readability score of 72 and a Flesch–Kincaid Grade Level of 6.5, making it accessible to most adults. The questionnaire format has demonstrated excellent test–retest reliability, with a weighted kappa of 0.98 (11).

#### QOLIBRI

The QOLIBRI questionnaire was utilized to assess QOL in study participants TBI. The QOLIBRI is a self-administered, disease-specific instrument comprising 37 items grouped into six domains: “Emotions,” “Feelings,” “Independence,” “Physical problems,” “Social relationships,” and “Thinking abilities” (6). Each item is rated by the respondent using a Likert-type scale, reflecting either satisfaction or the degree of being bothered by specific issues related to TBI. The QOLIBRI was administered in the paper format and participants were provided with standardized instructions and completed the questionnaire independently whenever possible. For individuals with cognitive or physical limitations that interfered with self-completion, a single trained research staff member aided the participant ensuring that the responses reflected the participant’s own perceptions (12). One participant required assistance from a trained research staff member to complete the QOLIBRI because of physical limitations; the staff member read the items and response options aloud, and the participant independently provided all responses.

### Statistical analysis

A mixed effects model was fit using maximum likelihood to evaluate the association between GOSE score and QOL from the QOLIBRI questionnaire. GOSE score was the primary independent variable of interest and one fixed effect of QOL. The GOSE scale covers of six levels of performance after TBI: (1) lower severe disability (GOSE 3); (2) upper severe disability (GOSE 4); (3) lower moderate disability (GOSE 5); (4) upper moderate disability (GOSE6); (5) lower good recovery (GOSE 7); and (6) upper good recovery (GOSE 8). GOSE score was transformed from its ordinal form to a continuous numerical form. This transformation involves the assumption that there is an equal amount of space between each level on the GOSE scale (i.e., that the difference between “lower severe disability” and “upper severe disability” is the same as the difference between “lower good recovery” and “upper good recovery.” This is an assumption that can be made for ordinal variables for clinical research with caution. An ANOVA test between results of the linear and ordinal models indicates that there is no statistical benefit to using the ordinal transformation in this context (AIC: linear = 7361.9, ordinal = 7368.7; χ^2^ = 1.19, *p* = 0.88). Further, the ordinal transformation of GOSE score substantially increases the number of parameters to fit on a sample of 138 individuals, introducing a concern for overfitting and difficulty of interpretation. Thus, we continue with the numeric transformation of GOSE score in the multivariate mixed-effects model and offer cautious interpretations.

The mixed-effects model also controlled for age, sex, injury severity, QOLIBRI domain, and an interaction term between GOSE and each domain as additional independent variables and as fixed effects. Participant ID is included as a random effect to account for the fact that each of the 138 participants contributed 6 domain scores (one per domain). QOLIBRI scores were derived from the six distinct domains. Each of these six domains are comprised further by five to seven individual questions ranked on five levels: (1) not at all, (2) slightly, (3) moderately, (4) quite, and (5) very. In the raw scores of the QOLIBRI questionnaire, directionality of outcomes varies by domain: higher scores in Emotions, Independence, Social relationships, and Thinking abilities reflect better QOL, as these items assess satisfaction within each domain. However, lower scores in the Feelings and Physical problems domains indicate *better* QOL, as these items measure symptom burden. To address this discrepancy, the scores for the Feelings and Physical problems domains were reverse coded so that for all domains, a higher score indicates a higher QOL. Individual item scores were then aggregated for each domain and standardized on a 0–100 scale using the official QOLIBRI scoring method so that scores among domains are directly comparable. Estimated marginal trends were calculated using the estimated fit of the mixed-effects model of GOSE within each QOLIBRI domain. All analyses were performed using R version 4.5.2 in RStudio and the lme4 and lmerTest packages.

## Results

Table 2 reports the mixed-effect regression results. Figure 1 illustrates the association between GOSE score and QOL stratified by the QOLIBRI domain, as estimated by the mixed-effects model. Table 3 reports the estimated marginal trends of GOSE score within each QOLIBRI domain. Significant independent variables correlated with QOL in the mixed-effects model included the Independence domain alone (*β* = −30.6, *p* = 0.003) compared to the reference category (Emotions). Age, sex, and injury severity (severe vs. moderate TBI) were not significantly correlated with QOL.

**Table 2:** Results of the mixed effects model using independent variables age, sex, GOSE score, severity of TBI, and QOLIBRI domain as fixed effects, correlated with QOL scores from the QOLIBRI survey.

Random effects	Variance	SD
Participant ID	0.0	0.0
Residual	417.2	20.4

Fixed effects	Estimate (Err)	95% CI	*p*-value
Age	−0.01 (8.9)	[−0.13, 0.11]	0.84
Sex			
Female	ref		
Male	−1.3 (1.8)	[−4.7, 2.2]	0.47
GOSE	−0.79 (1.2)	[−3.2, 1.5]	0.57
Severity of TBI			
Moderate	ref		
Severe	−1.2 (2.1)	[−5.8, 2.9]	0.50
Domain			
Emotions	ref		
Feelings	−14.1 (10.3)	[−34.4, 5.9]	0.17
Independence	−30.6 (10.3)	[−51.1, −10.8]	0.003**
Physical problems	11.5 (10.3)	[−8.2, 32.6]	0.26
Social relationships	12.6 (10.3)	[−6.7, 32.3]	0.22
Thinking abilities	−16.4 (10.3)	[−36.3, 3.9]	0.11
GOSE*Domain			
Emotions	ref		
Feelings	3.0 (1.6)	[0.10, 6.1]	0.057 (.)
Independence	11.2 (1.6)	[8.1, 14.5]	<0.001***
Physical problems	3.6 (1.6)	[0.38, 6.5]	0.025*
Social relationships	1.5 (1.6)	[−1.6, 4.7]	0.33
Thinking abilities	6.5 (1.6)	[3.3, 9.6]	<0.001***

Random effect participant ID is included to account for variation among survey participants. Coefficient estimates, standard errors, 95% confidence intervals, and *p*-values of each independent variable are reported. Variants, standard deviations, and residuals for random effects are reported.

**Figure 1:**
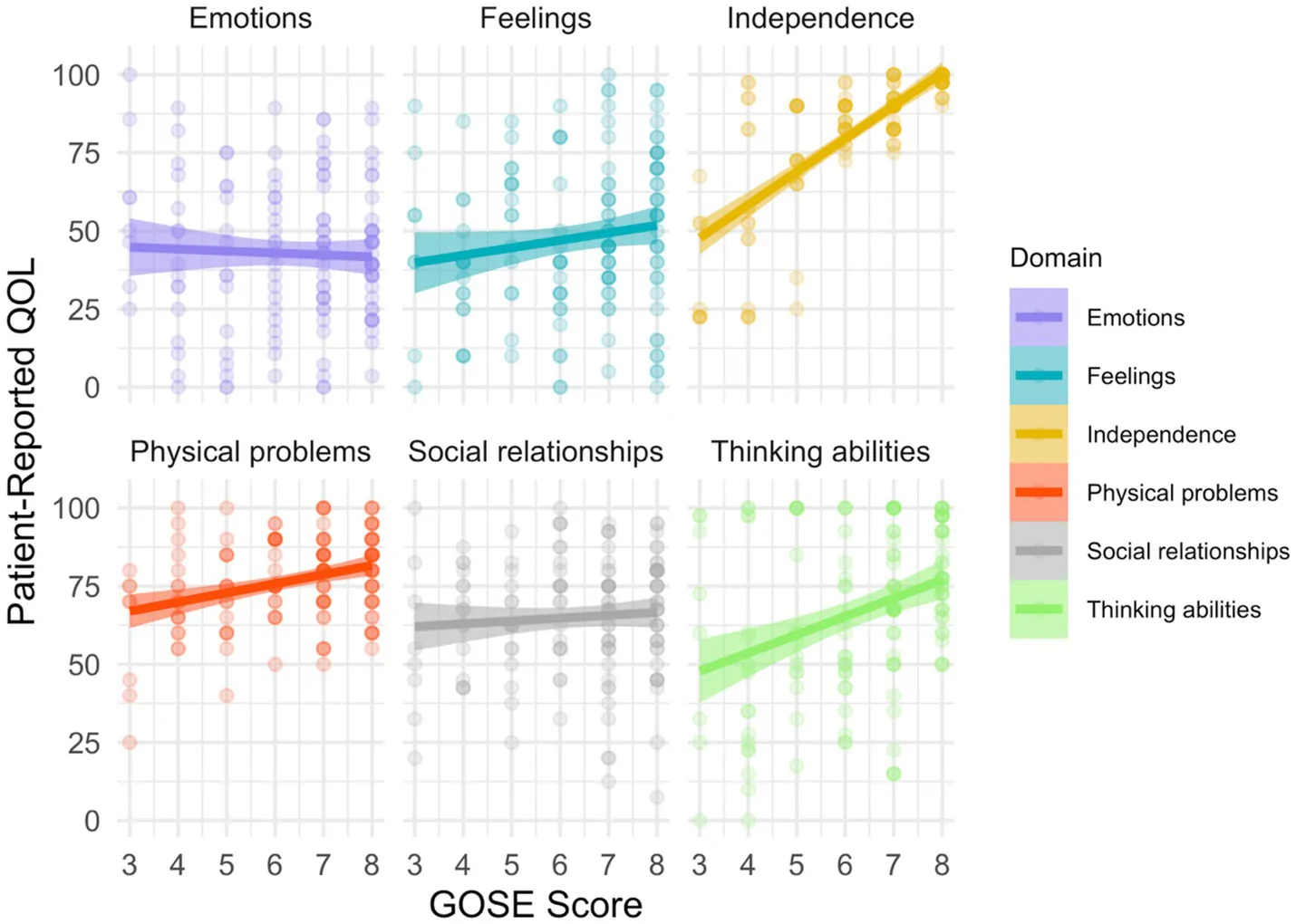
Visualization of the relationship between GOSE score and QOL scores (standardized from 0–100) stratified by QOLIBRI domain. Standard errors are illustrated for each relationship.

**Table 3:** Estimated marginal trends for the estimated coefficients of interaction terms (GOSE score with QOLIBRI domain) from the mixed-effects model.

Domain	GOSE trend (SE)	95% CI	*t*-value	*p*-value
Emotion	−0.79 (1.2)	[−3.1, 1.5]	−0.67	0.51
Feeling	2.2 (1.2)	[−0.10, 4.6]	1.9	0.061 (.)
Independence	10.4 (1.2)	[8.1, 12.7]	8.8	<0.001***
Physical	2.8 (1.2)	[0.44, 5.1]	2.3	0.019*
Social	0.74 (1.2)	[−1.6, 3.1]	0.63	0.53
Thinking	5.7 (1.2)	[3.3, 8.0]	4.8	<0.001***

The individual GOSE trend, standard error, 95% confidence interval, *t*-value, and *p*-value are reported, along with significance levels. Estimations for all domains were made on 802 degrees of freedom.

The interaction terms between GOSE and QOLIBRI domains and the estimated marginal trends for GOSE within each domain highlighted key findings. GOSE*Independence (*β* = 11.2, *p* < 0.001) and GOSE*Thinking abilities (*β* = 6.5, *p* < 0.001) were estimated as significantly different from the baseline Emotions domain (Table 2). Estimated marginal trends for GOSE within the Independence (*β* = 10.4, *p* < 0.001) and Thinking abilities (*β* = 5.7, *p* < 0.001) domains were strong, significant correlates of higher QOL (Table 3), indicating that functional recovery has the most significant association with the patients’ sense of autonomy and cognitive functioning. The interaction of GOSE*Physical problems (*β* = 3.6, *p* = 0.025) was also significantly different from the baseline domain, and GOSE within the Physical problems domain (*β* = 2.8, *p* = 0.019) indicates a significant association between increased GOSE score and increased QOL relative to physical problems (i.e., participants reported being less bothered by physical symptoms with higher functional recovery). Finally, the interaction between GOSE*Feelings (*β* = 3.0, *p* = 0.057) and estimated marginal trend of GOSE within Feelings (*β* = 2.2, *p* = 0.061) yielded weak but suggestive associations. Neither the interaction term for the Social relationships domain nor the estimated marginal trend for this domain were significant, indicating that QOL within the Social relationships domain was not significantly different from the reference (Emotions), and that the GOSE trend within Social relationships was also insignificant.

Participant ID, the sole random effect for this model, indicates a variance and standard deviation of zero, suggesting a singular result for this model and a residual result of 417.2 (SD = 20.4). The variance of zero suggests that this model detected no between-participant variability in QOL scores after accounting for all other independent variables in this model. Based on only these variables present in this model, there is little to no variation in domain-specific QOL on the individual level, and all variation instead lies in the residuals, which account for the unexplained variability in the model. Dividing each residual by the standard deviation to obtain standardized residuals, the scaled minimum is −2.9, median is −0.004, and maximum is 2.7, suggesting that the residuals are roughly centered symmetrically around zero and do not violate model assumptions related to normality and homoscedasticity. Further, to confirm the variance result as singular and not an artifact of the default modeling algorithm, we fit versions of the mixed-effects model with restricted maximum likelihood and the bound optimization by quadratic approximation (“bobyqa”), which both additionally yielded the singular result.

A sensitivity analysis was performed by fitting a generalized estimating equation (GEE) model on the same data to provide further confidence that the coefficient estimates, standard errors, and significance values estimated by the mixed-effects model were not just borne of the singular fit, but help strengthen the finding that there is no meaningful between-participant variation. This model accounts for repeated within-participant domain observations without depending on estimating variance. The results of the GEE model are reported in Supplementary Table 1 and show that the coefficient estimates are near-identical to those estimated by the fixed effects in the mixed-effects model, and the significance patterns are the same (Independence, GOSE*Independence, GOSE*Physical, and GOSE*Thinking are all statistically significant). The GEE reports an estimated within-participant correlation of near zero (*ɑ* = −0.059, standard error = 0.017), which supports the mixed-effects model’s finding of near-zero random effect variance.

## Discussion

This study found that the GOSE score alone does not significantly correlate with patient-reported QOL from the QOLIBRI questionnaire. Controlling for Participant ID as a random effect, there was no detected variation in between-patient outcomes, underscoring its limitations as a standalone indicator of recovery (Table 2). That is, there are additional explanatory variables unaccounted for in this model that could address unexplained residual variance that are not related to patient-reported GOSE scores. Even with the inclusion of additional independent variables such as age, sex, injury severity, QOLIBRI domains, and their interaction effects were included, GOSE score alone is not significantly associated with QOL. This finding highlights that functional status, although important, explains only part of the complex multidimensional experience of recovery after TBI.

The limited association between GOSE score alone and QOL is consistent with prior work (3, 9, 10, 13). Studies have reported a moderate-to-strong association between GOSE and health-related QOL but similarly conceptualized QOL as a single, global construct rather than examining domain-specific outcomes (3, 9, 14). By contrast, our domain-specific analysis extends these findings by demonstrating that the estimated marginal trends of GOSE within the Independence, Physical problems, and Thinking domains were the most strongly associated with improved QOL. Importantly, the absence of variation of between-person differences explained by the random effect, as indicated by the singular result (Participant ID, variance = 0, SD = 0) shows that most of the variation in QOL is captured by variation in self-reported QOLIBRI domain measures. This finding suggests that gains in functional independence such as returning to work, driving, or managing activities of daily living are critical for TBI survivors’ subjective well-being. Similarly, restoration of cognitive function, including attention and memory are just as critical to regaining autonomy and normalcy in academic and occupational settings. The intertwined nature of these domains is important to recognize, as declines in independence or cognition may adversely reinforce one another, creating a cycle that impedes recovery and reintegration into society negatively affecting QOL. However, the singular result along with the residual in the random effects results suggests that there are many more factors for which researchers can account in domain-dependent analyses of GOSE score and QOL. These will be briefly addressed in the limitations.

The raw scores in the Physical problems domain of QOLIBRI measures symptom burden (e.g., headaches, dizziness, fatigue), with lower scores indicating fewer symptoms and better QOL. With reverse coded and standardized scores used in the mixed-effects model, higher GOSE score is significantly associated with higher patient-reported QOL. It is reasonable to hypothesize that a higher functional recovery would significantly reduce physical burden, and the observed trend is consistent with clinical expectations. Although there is a significant association, physical symptom burden may also be associated with secondary factors. Post-head injury symptoms, chronic pain syndromes, medication adverse effects, and psychosomatic interactions may contribute to physical burden but may not be captured by functional status. In fact, it has been reported that unlike functional outcomes which show a clear dose–response association with TBI severity, TBI-related symptoms show elevated rates of persistence with sometimes conflicting associations with TBI severity (9, 15, 16).

The absence of significant associations between GOSE within the Feelings domain with QOL is also noteworthy. These domains capture constructs such as interpersonal relationships, social participation, and self-identity. Unlike independence and cognition, which improve in parallel with functional recovery, challenges with social reintegration and changes in self-perception may require targeted therapeutic approaches. These obstacles for TBI survivors may be associated with contextual factors such as social support networks, employment opportunities, cultural expectations, and psychiatric comorbidities (17–19). This echoes findings from TRACK-TBI and other large-scale prospective studies that have shown persistent symptom burden, emotional distress, and reduced QOL even among individuals with favorable GOSE scores (9). These observations underscore the importance of examining how functional recovery translates into each of the QOL domains, as functional improvement does not affect all domains equally or to the same degree. This emphasizes the need for critical distinction between functional and meaningful recovery, as the latter may require the integration of psychosocial and emotional support to improve QOL.

Interestingly, demographic variables did not emerge as significantly correlated with QOL. Neither older age nor male sex were associated with higher QOL scores in this sample. In this population, cognitive and physical deficits after TBI may interact synergistically to lead to loss of independence thus drastically reducing QOL (20). Our findings are likely reflecting sample-specific factors, considering a potential survival effect in which only relatively higher-functioning older survivors remained in the cohort. Though some studies do find that men report higher QOL than women in some prior reports of sex-based disparities in TBI outcomes (21), the mechanisms remain incompletely understood. The predominance of male participants (79.7%) warrant cautious interpretation given potential sample bias and skewed incidence of TBI in males. Notably, injury severity (moderate vs. severe TBI) was not significantly correlated with QOL for this sample. This finding is consistent with prior evidence suggesting that initial clinical severity indices, like the Glasgow Coma Scale, are not always reliable predictors of long-term QOL (22, 23). Thus, while severity remains essential for acute prognosis, its predictive value for long-term patient-reported QOL outcomes is limited (24).

The strength of the study is its use of validated, TBI-specific measures (GOSE and QOLIBRI) in a well-defined msTBI cohort, enabling mapping of functional recovery to QOL. Despite this strength, several limitations must be acknowledged. The cross-sectional design precludes conclusions regarding causality or the temporal sequence of recovery. Longitudinal data are needed to clarify whether improvements in GOSE are associated with subsequent gains in QOL, or whether QOL domains evolve independently over time. Future research should prioritize longitudinal studies that track both GOSE and QOLIBRI domains at multiple timepoints post-injury. Such data would illuminate trajectories of functional versus psychosocial recovery and identify critical periods for intervention. Second, the reliance on self-reported QOL introduces potential for recall bias and subjectivity. Although QOLIBRI is validated, responses may be associated with mood, cultural attitudes toward illness, or social desirability bias (12). Third, the study excluded individuals with GOSE scores of 1–2 (death or vegetative state), which is appropriate for self-report measures but restricts generalizability to the highest-severity cases. This may bias findings toward higher-functioning survivors and underestimate the burden of QOL impairment across the full spectrum of TBI.

From a clinical perspective, the findings underscore the necessity of multidimensional outcome assessment. Clinicians should recognize that “favorable outcome” defined by GOSE does not necessarily equate to QOL. By broadening the definition of recovery to include patient-reported QOL, clinicians and researchers can more accurately capture the true burden of TBI and design interventions that address the full spectrum of patient needs. Relying solely on functional scales such as GOSE risks overlooking persistent deficits in social participation, emotional well-being, and self-identity. Routine incorporation of patient-reported outcomes such as QOLIBRI into clinical care could facilitate individualized treatment planning and more accurately reflect recovery from the patient’s perspective.

## Conclusion

This study provides evidence that while GOSE is an important indicator of functional status after msTBI, it explains only a small proportion of variance in patient-reported QOL. Incorporating demographic variables and accounting for QOL domain substantially increases the proportion of variance explained by the model. Functional recovery appears to be most strongly associated with autonomy and cognitive functioning, whereas psychosocial domains are less directly tied to GOSE and require targeted intervention. These findings highlight the need for multidimensional outcome assessment that integrates both clinician-rated and patient-reported measures. Future work should expand on these findings through longitudinal designs and incorporation of psychosocial and biological variables, with the goal of developing prognostic models that capture both functional and meaningful recovery in TBI survivors.

## Permission to reuse and copyright

No copyrighted materials were used in the preparation of this manuscript. All text, figures, and tables are original and created by the authors. No material from other sources (including published works or web-based content) was reproduced, adapted, or reused; therefore, no permissions were required. All figures comply with Frontiers in Neurology figure preparation and submission guidelines.

## Data Availability

The raw data supporting the conclusions of this article will be made available by the authors, without undue reservation.
