# Supplementary material for: Functional recovery and domain-specific quality of life after moderate-to-severe traumatic brain injury: a mixed-effects analysis
Source: Front Neurol. 2026 Oct 27;17:1871168. doi: 10.3389/fneur.2026.1871168 (PMC13747861; doi:10.3389/fneur.2026.1871168)
Supplement: Supplementary file 1 [file Table_1.DOCX]

**Supplementary Table 1**. Side-by-side comparison of results of the fixed effects model (reported in the main text) and the generalized estimating equations model as a sensitivity analysis.

|  |  | **Mixed Effects Model** | | | | **Generalized Estimating Equations** | | |  |
| --- | --- | --- | --- | --- | --- | --- | --- | --- | --- |
| *Variables* | | *Estimate (Err)* | *95% CI* | *p-value* | *Estimate (Err)* | | *95% CI* | *p-value* | |
| *Age* | | -0.01 (8.9) | [-0.13, 0.11] | 0.84 | -0.01 (0.04) | | [-0.10, 0.075] | 0.78 | |
| *Sex* | |  |  |  |  | |  |  | |
|  | Female | ref | | | | ref | | |  |
|  | Male | -1.3 (1.8) | [-4.7, 2.2] | 0.47 | -1.3 (1.5) | | [-4.3, 1.7] | 0.41 | |
| *GOSE* | | -0.79 (1.2) | [-3.2, 1.5] | 0.57 | -0.77 (1.3) | | [-3.9, 1.5] | 0.39 | |
| *Severity of TBI* | |  |  |  |  | |  |  | |
|  | Moderate | ref | | | | ref | | |  |
|  | Severe | -1.2 (2.1) | [-5.8, 2.9] | 0.50 | -1.2 (1.4) | | [-3.4, 1.8] | 0.56 | |
| *Domain* | |  |  |  |  | |  |  | |
|  | Emotion | ref | | | | ref | | |  |
|  | Feeling | -14.1 (10.3) | [-34.4, 5.9] | 0.17 | -14.0 (12.7) | | [-38.8, 10.9] | 0.27 | |
|  | Independence | -30.6 (10.3) | [-51.1, -10.8] | 0.003** | -30.5 (14.2) | | [-58.3, -2.8] | 0.031* | |
|  | Physical | 11.5 (10.3) | [-8.2, 32.6] | 0.26 | 11.6 (9.3) | | [-6.7, 29.8] | 0.21 | |
|  | Social | 12.6 (10.3) | [-6.7, 32.3] | 0.22 | 12.7 (10.2) | | [-7.2, 32.7] | 0.21 | |
|  | Thinking | -16.4 (10.3) | [-36.3, 3.9] | 0.11 | -16.3 (17.4) | | [-50.4, 17.8] | 0.34 | |
| *GOSE*Domain* | |  |  |  |  | |  |  | |
|  | Emotion | ref | | | | ref | | |  |
|  | Feeling | 3.0 (1.6) | [0.10, 6.1] | 0.057 (.) | 3.0 (1.9) | | [-0.78, 6.8] | 0.12 | |
|  | Independence | 11.2 (1.6) | [8.1, 14.5] | <0.001*** | 11.2 (2.0) | | [7.2, 15.1] | <0.001*** | |
|  | Physical | 3.6 (1.6) | [0.38, 6.5] | 0.025* | 3.5 (1.4) | | [0.79, 6.3] | 0.011* | |
|  | Social | 1.5 (1.6) | [-1.6, 4.7] | 0.33 | 1.5 (1.6) | | [-1.5, 4.6] | 0.33 | |
|  | Thinking | 6.5 (1.6) | [3.3, 9.6] | <0.001*** | 6.4 (2.5) | | [1.5, 11.3] | 0.009** | |
